# Granulomatous Brain Involvement in Common Variable Immunodeficiency: A Case Report

**DOI:** 10.7759/cureus.67799

**Published:** 2024-08-26

**Authors:** Maria Rocha, Rita Gouveia, Ana Neves, Mariana Matos, Sergio Madureira

**Affiliations:** 1 Internal Medicine, Centro Hospitalar Universitário de São João, Porto, PRT

**Keywords:** case report, infiltrative brain lesions, granulomatous-lymphocytic interstitial disease, common variable immunodeficiency, autoimmunity

## Abstract

Common variable immunodeficiency (CVID) is a primary disorder characterized by impaired B cell differentiation and defective immunoglobulin production. This condition often presents with a wide range of clinical manifestations, including increased frequency and severity of infections, autoimmune diseases, and inflammatory disorders, which can lead to delays in diagnosis. Granulomatous involvement of the brain is an extremely rare but severe manifestation of CVID. We present a case of a woman in her 30s with a history of Evans syndrome and lymphocytic alveolitis who was admitted with persistent headache without neurological symptoms. Imaging revealed multiple infiltrative brain lesions. Despite the absence of recurrent infections, the patient’s history of autoimmune manifestations and immunoglobulin deficiencies led to the diagnosis of CVID without the need for a brain biopsy. Treatment with intravenous immunoglobulin and immunosuppressive therapy resulted in significant clinical improvement and resolution of brain lesions. This case highlights the importance of considering CVID in patients with autoimmune manifestations and the effectiveness of prompt immunoglobulin replacement and immunosuppression in managing severe presentations of this condition.

## Introduction

Common variable immunodeficiency (CVID) is a prevalent primary inborn immune disorder, with variable inheritance, affecting approximately 1 in 25,000 individuals [1]. It is characterized by impaired B cell differentiation, leading to defective immunoglobulin production. CVID is defined by markedly reduced serum concentrations of immunoglobulin G (IgG), usually below 400 mg/dL, along with at least one of the following: poor or absent response to immunization, low levels of immunoglobulin A (IgA) and/or immunoglobulin M (IgM), or the absence of other immunodeficiencies [2-4]. Patients with CVID have immune dysregulation, leading to an increased risk of bacterial infections and a higher incidence of autoimmune diseases, inflammatory disorders, and malignancies [2-4]. The term “variable” in CVID refers to the heterogeneous clinical manifestations of the condition, including chronic lung disease, gastrointestinal and liver disorders, and granulomatous infiltrations of various organs. Consequently, because of the broad clinical manifestations, the diagnosis of CVID is often delayed. Granulomatous infiltration of the brain is extremely rare, occurring in only 5% of patients with CVID-related granulomatous disease [5]. We present the case of a young woman with a history of Evans syndrome and lymphocytic alveolitis who developed severe granulomatous brain involvement, leading to a diagnosis of CVID.

## Case presentation

A caucasian 32-year-old woman was admitted to the emergency department with a left temporoparietal headache of an intensity 7/10 intensity that began three days prior. The pain was constant and increasingly unresponsive to analgesia. She did not report variation with head position or focal neurologic deficits. She also reported nausea and had two episodes of vomiting. She lived in an urban area without contact with animals, consumed only potable water, pasteurized dairy products, and well-cooked meat, and had an up-to-date vaccination record. She had no recent travel outside Europe and no contact with ill individuals. Her medical history included a diagnosis of Evans syndrome, characterized by autoimmune hemolytic anemia and immune thrombocytopenia, made five years before that was treated with corticosteroids. Two years before, she had also been evaluated for bilateral pulmonary infiltrates consistent with lymphocytic alveolitis (CD4/CD8 = 2.47) on bronchoalveolar lavage, with a biopsy showing lymphocytic infiltration without granulomas. On physical examination, she was hemodynamically stable (blood pressure: 126/73 mmHg, heart rate: 64 beats per minute), showed no signs of respiratory distress with an oxygen saturation of 98% on room air, and was afebrile. Her neurologic examination revealed no abnormalities, and the remainder of the physical examination findings were unremarkable.

**Figure 1:**
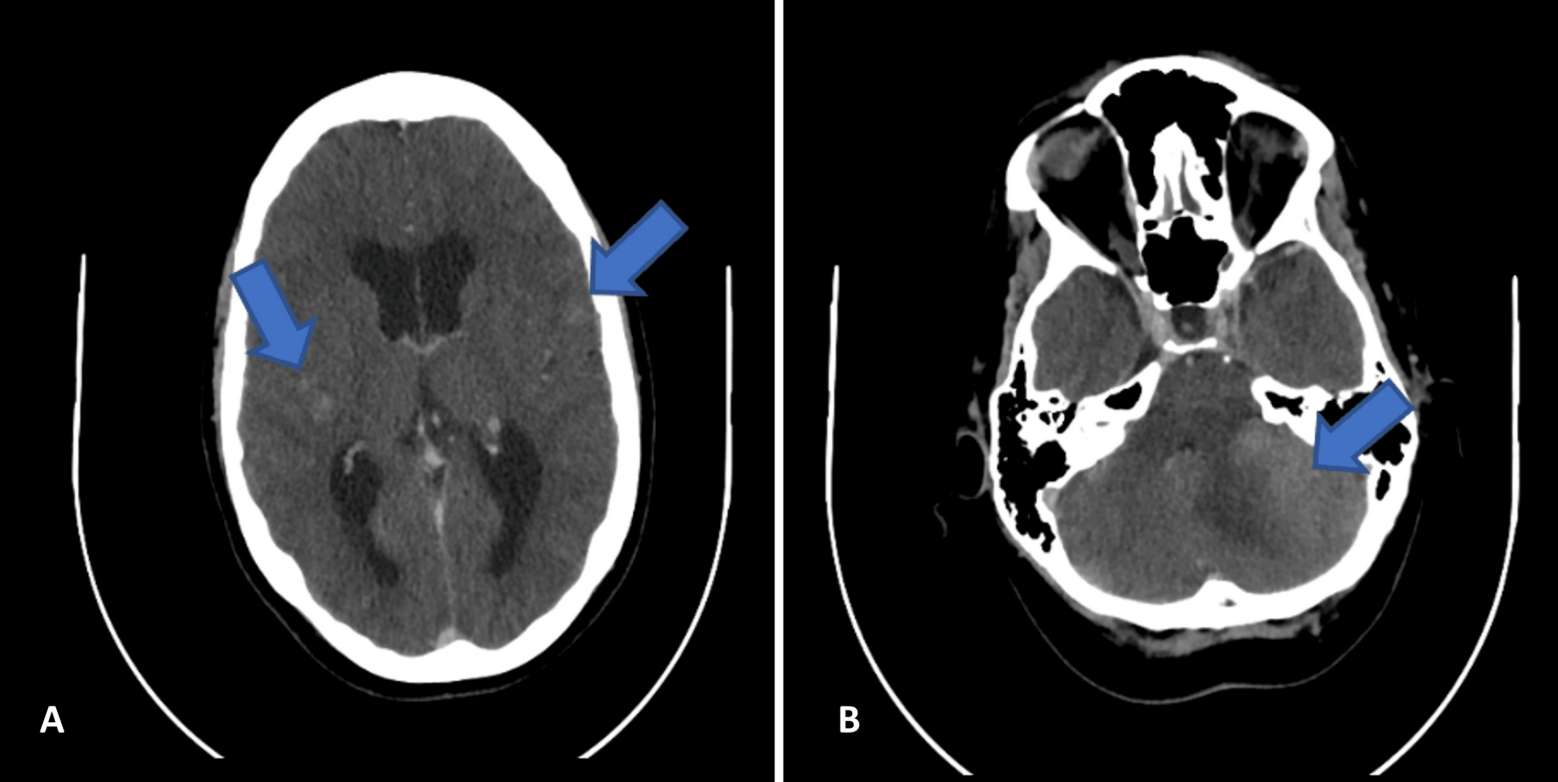
Brain CT scan in the emergency department. Blue arrows showing infiltrative (A, B) brain lesions. Abbreviation: CT, computed tomography.

A computed tomography (CT) scan of the brain with arterial and venous phase contrast was performed, along with general blood tests. The CT scan revealed multiple infiltrative lesions in the brain parenchyma, both infra and supratentorial, with a hyperdense appearance and contrast enhancement causing mass effect. The largest lesion was in the left cerebellar hemisphere (Figure 1). Blood tests showed mild thrombocytopenia (120,000/µL) and a mildly elevated C-reactive protein (6.6 mg/L), with no other abnormalities. 

Although she was not on immunosuppressive therapy at the time, her history of autoimmune disease suggested immunologic dysregulation. Therefore, a lumbar puncture was performed to rule out infection. Cerebrospinal fluid (CSF) analysis showed 48 cells (98% mononuclear), protein 0.7 g/L, glucose > 50% of serum value, and adenosine deaminase 3 U/L. Empiric therapy for central nervous system infection with ampicillin, ceftriaxone, and acyclovir was initiated, and she was admitted to the internal medicine ward for further investigation. On the first day, common infectious causes were excluded by direct Gram and India ink tests and polymerase chain reaction assessment for Listeria and Herpes simplex. Antimicrobial therapy was discontinued.

**Figure 2:**
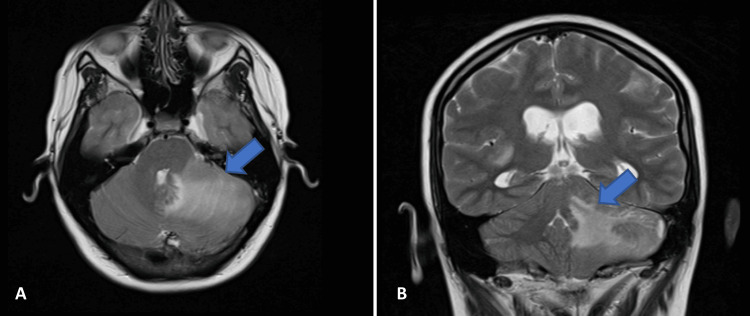
Brain MRI within the first few days after admission. The blue arrow (A, B) indicates the hypersignal in T2 FLAIR of the largest lesion in the left cerebellar hemisphere. Abbreviation: MRI, magnetic resonance imaging.

At this point, granulomatous brain disease or infiltration by hematologic malignancy was suspected. Magnetic resonance imaging (MRI) was performed to clarify the etiology of the brain lesions (Figure 2). Extended blood test results (Table 1) showed low levels of IgG (394 mg/dL), IgA (<6 mg/dL), and IgM (23 mg/dL), indicating a probable diagnosis of CVID, with previous Evans syndrome and lymphocytic interstitial lung disease, now presenting with granulomatous cerebral involvement. Immunophenotypic studies of blood and CSF showed a polyclonal population of B cells. A thoraco-abdominal-pelvic CT scan revealed bilateral pulmonary ground glass opacities, uniform hepatomegaly (16.6 cm), and splenomegaly (15.2 cm), but no lymphadenopathy or other findings.

**Table 1:** Blood tests performed for investigation. Abbreviation: NA, not applicable; anti-dsDNA, Anti-double-stranded deoxyribonucleic acid

Analyte	Patient Result	Reference Range
Hemoglobin (g/dL)	14.3	12-14
White blood cells (x10^9^/L)	5.95	4-11
Lymphocytes (x10^9^/L)	2.7	1-4
Platelets (x10^9^/L)	118	150-400
Erythrocyte sedimentation rate (mm/1ªh)	4	<20
Angiotensin converting enzyme (U/L)	30	<40
Thyroid-stimulating hormone (mU/L)	0.51	0.5-5
Antinuclear antibodies	Negative	NA
Anti-dsDNA antibodies	Negative	NA
Rheumatoid factor	Negative	NA
Anti-cyclic citrullinated peptide	Negative	NA
Anti-neutrophil cytoplasmic antibodies	Negative	NA
Immunoglobulin G (mg/dL)	464	600-1600
Immunoglobulin A (mg/dL)	<6	70-400
Immunoglobulin M (mg/dL)	17	50-300

**Figure 3:**
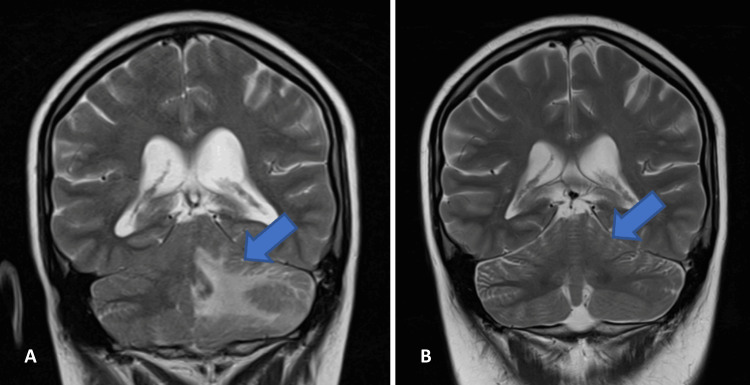
Comparative images of the brain MRI at admission (A) and one month after treatment initiation (B). The blue arrow indicates the infiltrative brain lesion. Abbreviation: MRI, magnetic resonance imaging.

Treatment was initiated with intravenous immunoglobulin (IVIG) replacement (500 mg/kg) and methylprednisolone (1 g for five days) followed by prednisolone (1 mg/kg/day). Co-trimoxazole (960 mg thrice weekly) was also started for *Pneumocystis jirovecii* prophylaxis. After 10 days of treatment, a follow-up brain CT showed a reduction in the size of the brain lesions and an improvement in vasogenic edema. She was discharged after 15 days with a schedule of IVIG (800 mg/kg per month) and a prednisolone tapering protocol. The patient reported no symptoms at a one-month follow-up, and the MRI was completely normal (Figure 3).

## Discussion

The broad range of manifestations associated with CVID and its indolent and variable presentation can delay the diagnosis, sometimes for several years. The condition can be diagnosed in childhood but more typically after puberty [2]. This clinical case evidenced a rare and severe form of CVID. This patient did not have a history of recurrent infections, but she had autoimmune manifestations for several years. Granulomatous disease is reported in 8% to 22% of patients, most commonly involving the lungs, lymph nodes, and spleen [3]. Brain involvement is extremely rare but can be life-threatening due to the risk of edema, hydrocephalus, and coma [5]. Given the severity of presentation in a patient with immunoglobulin deficiency and autoimmune manifestations (Evans syndrome and lymphocytic pneumonia), the diagnosis of CVID was assumed without a brain biopsy. The immunosuppressive therapy combined with immunoglobulin replacement was effective, leading to a total resolution of lesions at the one-month follow-up.

## Conclusions

We presented a case of an extremely rare manifestation of CVID with granulomatous brain involvement. Early recognition and diagnosis of CVID, despite its variable presentation, are critical for preventing life-threatening complications. This case underscores the importance of considering CVID in patients with autoimmune manifestations and immunoglobulin deficiency, even without recurrent infections. Timely initiation of treatment with intravenous immunoglobulin and immunosuppression can lead to significant clinical improvement and resolution of severe manifestations. Physicians should remain vigilant for atypical presentations of CVID to ensure prompt and effective management.
